# Disparities in access to and use of HIV-related health services in the Netherlands by migrant status and sexual orientation: a cross-sectional study among people recently diagnosed with HIV infection

**DOI:** 10.1186/s12879-019-4477-2

**Published:** 2019-10-29

**Authors:** Janneke P. Bil, Freke R. Zuure, Debora Alvarez-del Arco, Jan M. Prins, Kees Brinkman, Eliane Leyten, Ard van Sighem, Fiona Burns, Maria Prins

**Affiliations:** 1 Department of Infectious Diseases Research and Prevention, Public Health Service of Amsterdam, Amsterdam, the Netherlands; 2 Amsterdam Infection and Immunity Institute (AI&II), Amsterdam UMC (location AMC), University of Amsterdam, Amsterdam, the Netherlands; 3 Department of Internal Medicine, Amsterdam UMC (location AMC), University of Amsterdam, Amsterdam, the Netherlands; 4 National Centre for Epidemiology. Instituto de Salud Carlos III, Universidad Complutense de Madrid, Madrid, Spain; 5 Department of Internal Medicine, OLVG, Amsterdam, the Netherlands; 6 Department of Internal Medicine, Haaglanden Medisch Centrum, The Hague, The Netherlands; 7 Stichting HIV Monitoring, Amsterdam, The Netherlands; 8 Institute for Global Health, University College London, London, UK

**Keywords:** Migrants, HIV/AIDS, Health services, Epidemiology

## Abstract

**Background:**

Migrants often face barriers to accessing healthcare. We examined disparities in access to and use of HIV-related health services between migrant and non-migrant people recently diagnosed with HIV living in the Netherlands, taken into account sexual orientation. Also, we examined differences in experiences in living with HIV between these groups.

**Methods:**

We used a questionnaire and clinical data collected between July 2013 and June 2015 among migrant and non-migrant participants of the European cross-sectional aMASE (Advancing Migrant Access to health Services in Europe) study in the Netherlands. Using univariable logistic regression analyses, we compared outcomes on between migrants and non-migrants, stratified by sexual orientation (with non-migrant men having sex with men [MSM] as the reference group).

**Results:**

We included 77 migrant MSM, 115 non-migrant MSM, 21 migrant heterosexual men, 14 non-migrant heterosexual men and 20 migrant women. In univariable analyses, all heterosexual groups were less likely to ever have had an HIV-negative test before their diagnosis and were more likely to be diagnosed late than non-migrant MSM. All migrant groups were more likely to have experienced difficulties accessing general healthcare in the Netherlands and were less likely to have heard of post-exposure prophylaxis than non-migrant MSM. Migrants frequently reported uncertainty about their rights to healthcare and language barriers. Most (93%) participants visited a healthcare facility in the 2 years before HIV diagnosis but only in 41% an HIV test was discussed during that visit (no statistical difference between groups). Migrant heterosexuals were more likely to have missed appointments at their HIV clinic due to the travel costs than non-migrant MSM. Migrant MSM and women were more likely to have experienced HIV discrimination in the Netherlands than non-migrant MSM.

**Conclusion:**

Disparities in access to and use of HIV-related health services and experiences exist by migrant status but also by sexual orientation. Our data suggests heterosexual men and women may particularly benefit from improved access to HIV testing (e.g., through provider-initiated testing), while migrant MSM may benefit from improved access to HIV prevention interventions (e.g., pre-exposure prophylaxis).

## Background

Migrants represent a significant group in the HIV epidemic across Europe, including in the Netherlands [1–3]. An estimated 22,900 people were living with HIV in the Netherlands in 2017, with 89% diagnosed and linked to care, 92% of those in care were on combination antiretroviral therapy (cART) and of those on ART 95% were virally supressed [4]. Almost half (43%) of all HIV-positive people in care in the Netherlands in 2017 were born outside of the Netherlands [4] and data suggest that migrants are doing less well in the cascade of care. HIV-positive people originating from South-East Asia, sub-Saharan Africa, Surinam, the Caribbean or Latin America were more likely to enter clinical care with late-stage infection (clinical AIDS event or a CD4-count < 350 cells/mm^3^) or an advanced HIV infection (AIDS or CD4-count < 200 cells/mm^3^) than those of Dutch origin [5]. In the Netherlands, migrants are also more likely to have higher rates of lost to follow-up from HIV care [6], a longer time to virological suppression after combination antiretroviral therapy (cART) initiation, and higher risk of treatment failure [4] than those of Dutch origin. These data are in line with findings across the European Union/Economic Area [3] and suggest migrants face barriers in accessing and utilizing HIV health services. Hence, we need to better understand the specific barriers migrants face. Such data guide the development of strategies aimed at improving HIV prevention measures as well as optimising individual and public health outcomes.

The advancing Migrant Access to health Services in Europe (aMASE) study was set up to examine access to and use of HIV-related health services and identify structural, cultural and financial barriers to HIV prevention, diagnosis and treatment among several migrant groups living in Europe [7]. Results of the aMASE study suggest opportunities for HIV testing and prevention are still being missed among migrants living in Europe [8–10]. However, the extent to which this differs between migrants and non-migrants and according to their sexual orientation is not well known. Therefore, this study aims to examine differences in access to and use of HIV-related health services between migrants and non-migrants recently diagnosed in the Netherlands, taken into account their sexual orientation. In addition, we examined differences in experiences in living with HIV between these groups.

## Methods

### Study design and procedures

A cross-sectional study was conducted among migrant and non-migrant individuals living with HIV in the Netherlands. Migrants were included in the clinic survey of the aMASE study, as described in detail elsewhere [7]. In summary, the aMASE study was conducted between July 2013 and June 2015 in nine European countries. Migrants were included if they were diagnosed with HIV within 5 years of recruitment, aged > 18 years, foreign-born and resident in the country of recruitment for > 6 months, and able to complete, either alone or supported, a computer-assisted self or personal interview in any one of the 15 languages available. For the present study, we used data collected at all participating HIV outpatient treatment clinics in the Netherlands (Amsterdam UMC [location AMC], OLVG in Amsterdam and Haaglanden Medisch Centrum, in The Hague). In addition to the aMASE data collection among migrants, in the Netherlands all non-migrants (i.e., those born in the Netherlands) who attended any of the three HIV outpatient treatment clinics during the same study period and were diagnosed with HIV within 5 years and aged > 18 years were also asked to participate.

Participants completed a questionnaire on HIV-related services (which included access to HIV testing and healthcare pre-diagnosis and access to treatment and ongoing care after HIV diagnosis) and experiences. Clinical data were obtained from the national ATHENA (AIDS Therapy Evaluation in the Netherlands) HIV cohort database [4]. For people who declined to participate, we collected data on age, country of birth, sexual orientation and reason for decline.

### Variables

#### Socio-demographic characteristics and migration history

Characteristics included self-reported sexual orientation, self-defined ethnicity, educational level, current work status, income level, age, household hunger in the past 4 weeks and attending religious services. Migration history included years since migration, age at migration, region of birth and immigration status.

#### Access to HIV testing and healthcare pre-diagnosis

We measured factors related to HIV diagnosis, HIV testing behaviour, access to care and awareness of post-exposure prophylaxis (PEP). Factors related to HIV diagnosis included: age at HIV diagnosis, years since HIV diagnosis, location of HIV diagnosis, reason for HIV test, CD4 count and late stage HIV infection at HIV diagnosis. Among migrants we also collected data on the years between migration to the Netherlands and HIV diagnosis, country of HIV diagnosis and country of previous HIV negative test. Factors related to HIV test behaviour included: ever had an HIV test before HIV diagnosis and years between previous negative HIV test and HIV diagnosis. Variables measuring access to care included registration at a general practitioner (GP) in the Netherlands (GPs are the first point of access to healthcare in the Netherlands), healthcare usage in the Netherlands in the 2 years before HIV diagnosis and experienced difficulties accessing healthcare in the Netherlands. Among those who used healthcare in the Netherlands, we asked which healthcare professionals were visited and whether or not an HIV test was discussed during these visits.

#### Access to treatment and ongoing care

We measured use of cART, time between HIV diagnosis and starting cART, self-reported cART adherence and self-reported difficulty to take HIV medication on a regular basis. Furthermore, participants were asked if they ever missed an appointment at the HIV clinic due to the travel costs.

#### Experiences in living with HIV

Participants were asked if they had disclosed their HIV status to their steady partner or to friends and family, if they received HIV support through any non-governmental organizations (NGOs), and if they ever felt discriminated in the Netherlands because of their HIV status, ethnicity, race or origin, or sexuality.

### Statistical analyses

As previous studies have shown outcomes are influenced by sexual orientation [8, 9], participants were grouped into: migrant MSM, non-migrant MSM, migrant heterosexual men, non-migrant heterosexual men, migrant women and non-migrant women. Socio-demographic characteristics and non-dichotomous outcomes were compared between groups using chi-square tests and Fisher’s exact tests for categorical variables and one-way ANOVA (normally distributed) and Kruskal Wallis tests (not normally distributed) for continuous variables. Dichotomous outcomes related to access to HIV prevention, testing, care pre-diagnosis, treatment, ongoing care and experiences in living with HIV were used as separate end-points. The univariable odds ratios (ORs) were calculated using logistic regression or penalized logistic regression in a table with a zero cell count [11].

In additional analyses, we adjusted outcomes for participants’ age by constructing multivariable models as age is a potential confounder. We also compared all outcomes by region of birth in MSM only, due to the low numbers of migrant heterosexual men and women.

In the analyses, participants with unknown or missing data were excluded. Analyses were performed using STATA Intercooled 13.1 (STATA Corporation, College Station, Texas, USA). A *p* value of < 0.05 was considered statistically significant.

## Results

Of 417 invited HIV-positive patients, 60% (*n* = 252) participated. The response rate was lower among migrants from Latin America/Caribbean and women and heterosexual men than non-migrants and MSM, respectively. Also, participants recruited at the Amsterdam UMC were less likely to participate than those recruited elsewhere.

In total 247 participants were included in the analyses (Table 1): 77 migrant MSM, 115 non-migrant MSM, 21 migrant heterosexual men, 14 non-migrant heterosexual men and 20 migrant women. Five non-migrant women were excluded from the analyses because of their low number. Groups differed with regard to variables reflecting socio-economic status (all *p*-values < 0.01), current age (*p* = 0.002), age at migration (*p* = 0.003), self-defined ethnicity (*p* < 0.001), immigration status (*p* < 0.001), attendance of religious services (*p* < 0.001) and recruitment site (*p* = 0.012) (Table 1). There was a difference in region of birth between MSM and heterosexual migrants (*p* < 0.001); with 40% of the MSM migrants originating from Europe and more than half of the heterosexual migrants originate from sub-Saharan Africa.

**Table 1:** Socio-demographic characteristics and migration history of Dutch aMASE-study participants (*n* = 247), 2013–2015

	Total		Migrant MSM		Non-migrant MSM		Migrant heterosexual men		Non-migrant heterosexual men		Migrant women		
	(*n* = 247)		(*n* = 77)		(*n* = 115)		(*n* = 21)		(*n* = 14)		(*n* = 20)		
	n/N	%	n/N	%	n/N	%	n/N	%	n/N	%	n/N	%	*p*-value
Higher educational level (college degree or higher)	107/247	43.3	33/77	42.9	61/115	53.0	4/21	19.0	6/14	42.9	3/20	15.0	0.003
Currently working	185/246	75.2	58/77	75.3	99/115	86.1	10/21	47.6	9/14	64.3	9/19	47.4	< 0.001
Lower income level (less than minimum wage)	91/231	39.4	36/68	52.9	16/113	14.2	18/19	94.7	6/14	42.9	15/17	88.2	< 0.001
Moderate/severe household hunger in the past 4 weeks	49/244	20.1	15/74	20.3	15/115	13.0	9/21	42.9	2/14	14.3	8/20	40.0	0.004
Age (Median, IQR)	41	33–49	36	28–46	43	36–51	45	40–48	41	33–55	39	33–48	0.002
Years since migration to the Netherlands (Median, IQR)	10	4–23	8	4–25	NA		11	7–15	NA		11	4–22	0.965
Age at migration to the Netherlands (Median, IQR)	25	21–33	24	21–29	NA		34	26–38	NA		24	19–39	0.003
Region of birth													< 0.001
Sub-Saharan Africa	30/118	25.4	4/77	5.2	NA		16/21	76.2	NA		10/20	50.0	
Latin America / Caribbean	27/118	22.9	19/77	24.7			3/21	14.3			5/20	25.0	
Europe	33/118	28.0	31/77	40.3			0/21	0.0			2/20	10.0	
Other	28/118	23.7	23/77	29.9			2/21	9.5			3/20	15.0	
Self-defined ethnicity													< 0.001^a^
European	149/245	60.80	35/76	46.1	99/114	86.8	0/21	0.0	12/14	85.7	3/20	15.0	
Other	96/245	39.20	41/76	54.0	15/114	13.2	21/21	100.0	2/14	14.3	17/20	85.0	
African	32/245	13.10	4/76	5.3	1/114	0.9	17/21	81.0	0/14	0.0	10/20	50.0	
American	6/245	2.40	3/76	3.9	0/114	0.0	0/21	0.0	0/14	0.0	3/20	15.0	
Asian	17/245	6.90	10/76	13.2	3/114	2.6	1/21	4.8	1/14	7.1	2/20	10.0	
Mixed	20/245	8.20	10/76	13.2	7/114	6.1	1/21	4.8	0/14	0.0	2/20	10.0	
Latin America / Caribbean	13/245	5.30	9/76	11.8	3/114	2.6	0/21	0.0	1/14	7.1	0/20	0.0	
Middle Eastern	8/245	3.30	5/76	6.6	1/114	0.9	2/21	9.5	0/14	0.0	0/20	0.0	
Immigration status													< 0.001
Permanent residency permit	84/108	77.8	63/72	87.5	NA		9/19	47.4	NA		12/17	70.6	
Temporary residency permit	16/108	14.8	8/72	11.1			4/19	21.1			4/17	23.5	
Refugee status / unknown	8/108	7.4	1/72	1.4			6/19	31.6			1/17	5.9	
Attending religious services at least once a year	74/239	31.0	23/72	31.9	20/115	17.4	14/18	77.8	2/14	14.3	15 /20	75.0	< 0.001
Recruitment site													0.012
Amsterdam UMC (location AMC), Amsterdam	76/247	30.8	26/77	33.8	30/115	26.1	8/21	38.1	6/14	42.9	6/20	30.0	
OLVG, Amsterdam	121/247	49.0	35/77	45.5	68/115	59.1	10/21	47.6	2/14	14.3	6/20	30.0	
Haaglanden Medisch Centrum, The Hague	50/247	20.2	16/77	20.8	17/115	14.8	3/21	14.3	6/14	42.9	8/20	40.0	

*MSM* men who have sex with men, *IQR* interquartile range, *NA* Not applicable

^a^ *p*-value for difference between European and Other

### Access to HIV testing and healthcare pre-diagnosis

The median age at HIV diagnosis was 39 years (IQR 31–47) and median time since HIV diagnosis was 2 years (IQR 1–4) (Table 2). Migrants were a median of 8 years (IQR 2–21) in the Netherlands before HIV diagnosis.

**Table 2:** Access to HIV testing and healthcare pre-diagnosis among Dutch aMASE-study participants (*n* = 247), 2013–2015

	Total		Migrant MSM		Non-migrant MSM		Migrant heterosexual men		Non-migrant heterosexual men		Migrant women		
	(*n* = 247)		(*n* = 77)		(*n* = 115)		(*n* = 21)		(*n* = 14)		(*n* = 20)		
	n	%	n	%	n	%	n	%	n	%	n	%	*p*-value^a^
Age (years) at HIV diagnosis (Median, IQR)^b^	39	31–47	34	26–44	41	33–49	42	38–47	38	32–50	37	30–46	0.006
Years since HIV diagnosis (Median, IQR)	2	1–4	2	1–3	3	1–4	2	1–3	3	1–4	2	1–4	0.046
Location of HIV diagnosis													< 0.001^c^
Sexual health clinic / HIV testing clinic	103/238	43.3	42/70	60.0	52/115	45.2	2/19	10.5	3/14	21.4	4/20	20.0	
Hospital	66/238	27.7	13/70	18.6	25/115	21.7	10/19	52.6	9/14	64.3	9/20	45.0	
GP	57/238	23.9	12/70	17.1	34/115	29.6	4/19	21.1	1/14	7.1	6/20	30.0	
Other^d^	12/238	5.0	3/70	4.3	4/115	3.5	3/19	15.8	1/14	7.1	1/20	5.0	
Reason for HIV test^e^													
It was part of a routine health checkup	89/245	36.3	30/77	39.0	51/114	44.7	4/21	19.1	3/13	23.1	1/20	5.0	
A doctor advised me to test due to health problems	86/245	35.1	18/77	23.4	36/114	31.6	13/21	61.9	5/13	38.5	14/20	70.0	
I felt I was at risk	48/245	19.6	22/77	28.6	21/114	18.4	3/21	14.3	1/13	7.7	1/20	5.0	
I had sexual contact with someone I knew/thought had HIV	37/245	15.1	19/77	24.7	13/114	11.4	0/21	0.0	3/13	23.1	2/20	10.0	
Other^f^	16/245	6.5	3/77	3.9	5/114	4.4	3/21	14.3	2/13	15.4	3/20	15.0	
Years between migration to the Netherlands and HIV diagnosis	8	2–21	6	1–22	NA		9	4–15	NA		10	2–19	0.911
Country of HIV diagnosis													0.696
The Netherlands	109/118	92.4	70/77	90.9	NA		19/21	90.5	NA		20/20	100.0	
Country of birth	7/118	5.9	5/77	6.5			2/21	9.5			0/20	0.0	
Other country	2/118	1.7	2/77	2.6			0/21	0.0			0/20	0.0	
CD4 cell count (cells/mm^3^) at HIV diagnosis (Median, IQR)^g^	400	220–570	430	280–580	475	320–647	125	40–230	225	20–570	260	50–430	< 0.001
Late-stage HIV infection at HIV diagnosis (AIDS or a CD4 count < 350 cells/mm3)	101/239	42.3	26/74	35.1	37/112	33.0	17/20	85.0	10/14	71.4	11/19	57.9	
Ever had a negative HIV test before HIV diagnosis	192/247	77.7	69/77	89.6	96/115	83.5	11/21	52.4	4/14	28.6	12/20	60.0	
Years between previous negative HIV test and HIV diagnosis (Median, IQR)^h,i^	2	1–4	2	1–4	1	0–3	6	1–9	6	2–11	5	3–12	0.002
Country of previous HIV negative test^i^													
The Netherlands	52/86	60.5	41/66	62.1	NA		5/10	50.0	NA		6/10	60.0	
Another country	34/86	39.5	25/66	37.9			5/10	50.0			4/10	40.0	
Registered at a GP in the Netherlands	241/247	97.6	73/77	94.8	115/115	100.0	19/21	90.5	14/14	100.0	20/20	100.0	
Healthcare usage in the Netherlands 2 years before HIV diagnosis^j^	202/217	93.1	52/56	92.9	110/115	95.7	15/17	88.2	12/14	85.7	13/15	86.7	
No HIV testing discussed during healthcare attendance in the 2 years before HIV diagnosis^k^	116/197	58.9	26/51	51.0	58/107	54.2	11/15	73.3	11/11	100.0	10/13	76.9	
Experienced difficulties accessing healthcare in the Netherlands	26/246	10.6	16/77	20.8	3/115	2.6	3/20	15.0	0/14	0.0	4/20	20.0	
Type of difficulties experienced in accessing healthcare in the Netherlands^e,l^													
I am still unsure of my rights to access healthcare	10/26	38.5	5/16	31.3	0/3	0.0	3/3	100.0	0/0	0.0	2/4	50.0	
Clinic opening hours are inconvenient	5/26	19.2	4/16	25.0	1/3	33.3	0/3	0.0	0/0	0.0	0/4	0.0	
There are long waiting times for an appointment	6/26	23.1	4/16	25.0	1/3	33.3	0/3	0.0	0/0	0.0	1/4	25.0	
I have difficulty communicating with staff because of language differences	8/26	30.8	3/16	18.8	0/3	0.0	2/3	66.7	0/0	0.0	3/4	75.0	
Other	13/26	50.0	8/16	50.0	3/3	100.0	1/3	33.3	0/0	0.0	1/4	25.0	
Ever heard of post-exposure prophylaxis (PEP)	162/245	66.1	46/77	59.7	101/113	89.4	3/21	14.3	8/14	57.1	4/20	20.0	

*MSM* men who have sex with men, *IQR* interquartile range, *GP* General Practitioner

^a^ only *p*-values are presented for variables not included in Fig. 2

^b^ 3 missings

^c^ *p*-value calculated without the other category

^d^ Other includes: antenatal care (*n* = 3), refugee center (*n* = 3), fertility clinic (*n* = 1), dentist (*n* = 1), self-test (*n* = 1), medical examination (*n* = 1), private clinic (*n* = 1), unknown (*n* = 1)

^e^ Total number and percentage exceeds 100% because more than one answer could be given

^f^ Other reasons are for example: pregnancy, test done without permission of participant, relationship, sexual assault

^g^ 8 missings

^h^ 56 missings

^i^ Only participants were included who had a previous negative HIV test before diagnosis

^j^ Only participants were included who lived in the Netherlands for 2 years or more and who were diagnosed with HIV in the Netherlands

^k^ Only participants were included who lived in the Netherlands for 2 years or more, who were diagnosed with HIV in the Netherlands and who had used healthcare in the Netherlands in the previous 2 years before HIV diagnosis

^l^ Only participants were included who experienced difficulties accessing healthcare in the Netherlands

While MSM were most frequently diagnosed at a sexual health or HIV testing clinic and most commonly tested for HIV because it was part of a routine health check-up, most heterosexuals men and women (both migrants and non-migrants) were diagnosed in a hospital and stated a doctor advised the HIV test due to health problems.

**Fig. 1:**
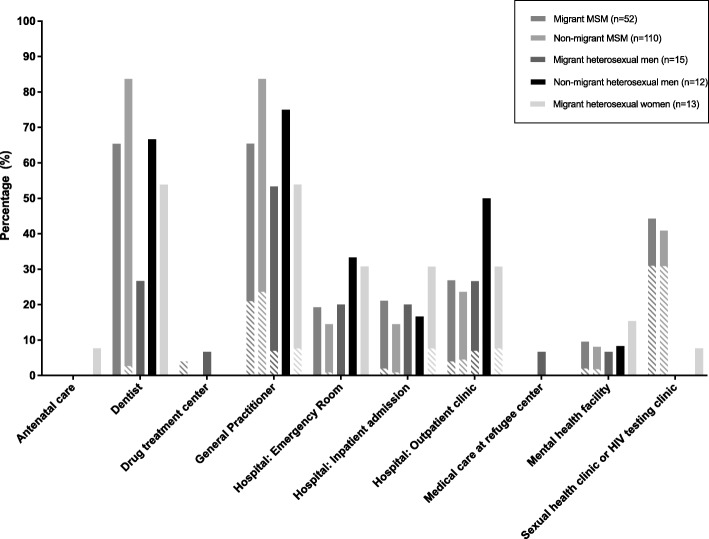
Healthcare attendance in the 2 years before HIV diagnosis and the proportion in which HIV testing was discussed among Dutch aMASE-study participants, 2013–2015. Note: dashed lines represent the proportion of cases in which an HIV test was discussed during healthcare attendance. MSM: Men who have sex with men

Overall, 78% had a previous negative HIV test before HIV diagnosis and 42% were diagnosed with a late-stage HIV infection. Most (93%) participants visited a healthcare facility in the 2 years prior to diagnosis, with 41% reporting that HIV testing was discussed (Table 2). Most participants who used a healthcare facility visited a GP and/or dentist; 26% of those who visited a GP, recalled that an HIV test was discussed (Fig. 1). The proportion visiting a GP was lower among migrant groups (range 53–65%) than among the non-migrant groups (range 74–84%) (*p* = 0.009).

**Fig. 2:**
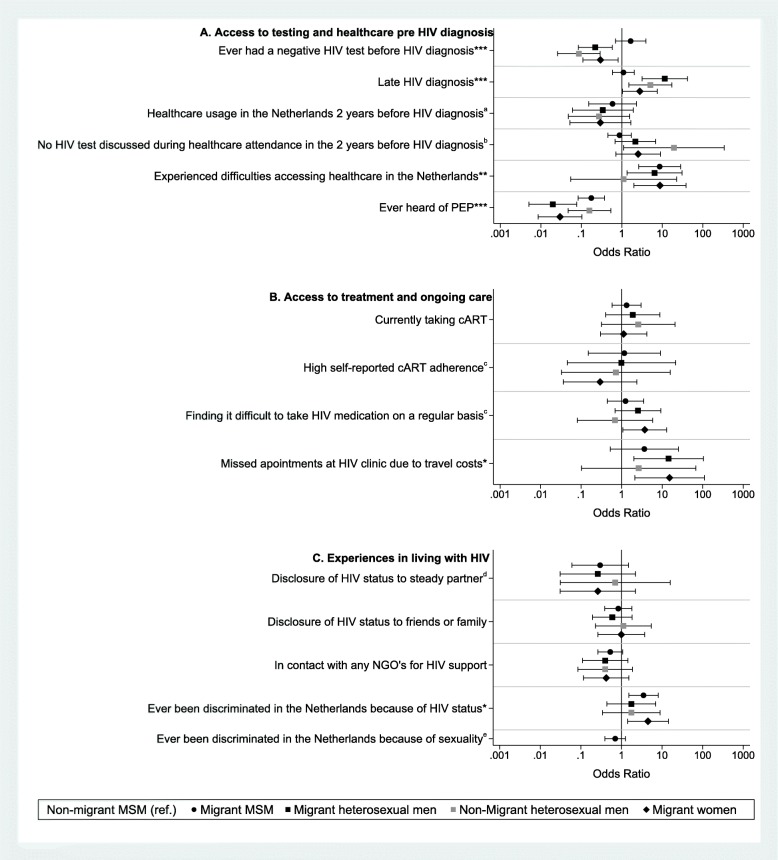
Univariable analyses of the relationship between migrant status and sexual orientation and (**a**) access to testing and healthcare pre HIV diagnosis, (**b**) access to treatment and ongoing care and (**c**) experiences in living with HIV among Dutch aMASE-study participants, 2013–2015. MSM: men who have sex with men; PEP: post-exposure prophylaxis; cART: combination antiretroviral therapy; NGO: non-governmental organisation. ^a^ Only participants were included who lived in the Netherlands for 2 years or more and who were diagnosed with HIV in the Netherlands. ^b^ Only participants were included who lived in the Netherlands for 2 years or more, who were diagnosed with HIV in the Netherlands and who had used healthcare in the Netherlands in the previous 2 years before HIV diagnosis. ^c^ Only participants were included who were currently using cART. ^d^ Only participants were included who had a steady partner. ^e^ Only MSM were included in this analysis. * *p* < 0.05 ** *p* < 0.01 *** *p* < 0.001

In univariable analyses (Fig. 2a), having had a previous HIV-negative test was less likely in migrant heterosexual men (OR:0.22, 95%-CI:0.08–0.58), non-migrant heterosexual men (OR:0.09, 95%-CI:0.03–0.29) and migrant women (OR:0.30, 95%-CI:0.11–0.81) than in non-migrant MSM, whereas the difference between migrant (OR:1.65, 95%-CI:0.70–3.91) and non-migrant MSM was not statistically significant. Also, among those who had an HIV-negative test, the median time between the previous HIV-negative test and HIV diagnosis was significantly longer among all heterosexual men and women than migrant and non-migrant MSM (*p =* 0.002, Table 2). Being diagnosed with a late-stage HIV infection was more likely in migrant heterosexual men (OR:11.49, 95%-CI 3.17–41.69), non-migrant heterosexual men (OR:5.07, 95%-CI:1.49–17.24) and migrant women (OR:2.79, 95%-CI:1.03–7.52) than non-migrant MSM, whereas this did not differ between migrant (OR:1.10, 95%-CI:0.59–2.04) and non-migrant MSM. All three migrant groups (MSM [OR:8.62, 95%-CI:2.61–28.48], heterosexual men [OR:6.43, 95%-CI 1.34–30.74] and women [OR:8.77, 95%-CI 1.98–38.97]) were more likely to have experienced difficulties accessing healthcare in the Netherlands than non-migrant MSM. Most frequently reported difficulties accessing healthcare in the Netherlands among migrants were uncertainty regarding their right to access healthcare and language barriers (Table 2). Overall, 66% had ever heard of PEP. Migrant MSM (OR:0.18, 95%-CI:0.08–0.37), migrant heterosexual men (OR:0.02, 95%-CI:0.01–0.08), non-migrant heterosexual men (OR:0.16, 95%-CI:0.05–0.53) and migrant women (OR:0.03, 95%-CI:0.01–0.10) were less likely to have heard of PEP than non-migrant MSM. Healthcare usage in the 2 years before HIV diagnosis and the proportion in which an HIV test was discussed during healthcare attendance did not differ significantly between groups.

Comparing outcomes by region of birth and restricting the analyses to MSM, MSM born in sub-Saharan Africa (OR:12.44, 95%-CI:0.98–157.33), another country in Europe (OR:8.96, 95%-CI:2.10–38.28) or in another region (OR:19.91, 95%-CI:4.75–83.38) were more likely to have difficulties accessing healthcare in the Netherlands than non-migrant MSM. Also, MSM born in sub-Saharan Africa (OR:0.12, 95%-CI:0.02–0.92), Latin America/Caribbean (OR:0.13, 95%-CI:0.04–0.39), another country in Europe (OR:0.29, 95%-CI:0.11–0.77) or in another region (OR:0.13, 95%-CI:0.05–0.36) were less likely to have heard of PEP than non-migrant MSM. No significant differences were found in the other outcomes on access to HIV testing and healthcare pre-diagnosis between region of birth among MSM.

### Access to treatment and ongoing HIV care

Overall, 86% of participants reported they were on cART, median time between HIV diagnosis and starting cART was 6 weeks (IQR 3–43). Almost all (98%) reported high cART adherence, 14% found it difficult to take HIV medication on a regular basis; 4% missed appointments at their HIV clinic due to the travel costs (Table 3).

**Table 3:** Access to treatment and ongoing HIV care among Dutch aMASE-study participants (*n* = 247), 2013–2015

	Total		Migrant MSM		Non-migrant MSM		Migrant heterosexual men		Non-migrant heterosexual men		Migrant women		
	(*n* = 247)		(*n* = 77)		(*n* = 115)		(*n* = 21)		(*n* = 14)		(*n* = 20)		
	n	%	n	%	n	%	n	%	n	%	n	%	*p*-value^a^
Currently taking cART	212/247	85.8	67/77	87.0	96/115	83.5	19/21	90.5	13/14	92.9	17/20	85.0	
Weeks between start cART and HIV diagnosis (Median, IQR)^b, c^	6	3–43	9	3–31	7	4–59	3	1–7	3	2–7	3	2–34	0.002
Reason for no cART use^d^													
My doctor says I do not need them yet	21/35	60.0	6/10	60.0	12/19	63.2	0/2	0.0	1/1	100.0	2/3	66.7	
I’m afraid of the side effects	3/35	8.6	0/10	0.0	2/19	10.5	0/2	0.0	0/1	0.0	1/3	33.3	
I’m on a treatment break I agreed with my doctor	5/35	14.3	1/10	10.0	4/19	21.1	0/2	0.0	0/1	0.0	0/3	0.0	
I will start soon/today	8/35	22.9	3/10	30.0	3/19	15.8	2/2	100.0	0/1	0.0	0/3	0.0	
I am waiting for approval	1/35	2.9	0/10	0.0	1/19	5.3	0/2	0.0	0/1	0.0	0/3	0.0	
High self-reported cART adherence^b, e^	205/209	98.1	65/66	98.5	93/95	97.9	18/18	100.0	13/13	100.0	16/17	94.1	
Finding it difficult to take HIV medication on a regular basis^b, f^	27/191	14.1	7/53	13.2	10/92	10.9	4/17	23.5	1/13	7.7	5/16	31.3	
Missed appointments at HIV clinic due to the travel costs	10/246	4.1	3/76	3.9	1/115	0.9	3/21	14.3	0/14	0.0	3/20	15.0	

*MSM* men who have sex with men, *cART* combination antiretroviral therapy, *IQR* interquartile range

^a^ only *p*-values are presented for variables not included in Fig. 2

^b^ Only participants were included who were currently using cART

^c^ 16 missings

^d^ Only participants were included who were not currently using cART. Total number and percentage exceeds 100% because participants could indicate more than one reason

^e^ Measured on a 4-point Likert scale and dichotomized for analyses whereas the answers strongly agree and agree on the statement “I always follow my doctor’s instructions about taking my HIV medication” represent high self-reported cART adherence and strongly disagree and disagree represents low self-reported adherence

^f^ Measured on a 4-point Likert scale and dichotomized for analyses whereas the answers strongly agree and agree on the statement “I find it difficult to take my HIV medication on a regular basis” represent finding it difficult to take HIV medication on a regular basis and strongly disagree and disagree represents not finding it difficult to take HIV medication on a regular basis

In univariable analyses (Fig. 2b), migrant heterosexual men (OR:14.44,95%-CI:2.00–104.05) and migrant women (OR:15.27,95%-CI:2.11–110.33) were more likely to have missed appointments at their HIV clinic due to the travel costs than non-migrant MSM. All heterosexual groups were more likely to start cART earlier after their HIV diagnosis than non-migrant and migrant MSM (*p* = 0.002, Table 3). No significant differences were found in the other outcomes on access to treatment and ongoing HIV care and there were no significant differences between region of birth among MSM.

### Experiences in living with HIV

Most participants had disclosed their HIV status to their steady partner (94%) and to friends and family (83%) and 23% were in contact with an NGO for HIV support (Table 4). 16% respondents reported experiencing discrimination because of their HIV status. Among migrants, 43% reported discrimination in the Netherlands because of their ethnicity, race or country of origin and 46% of migrant and non-migrant MSM reported discrimination because of their sexuality.

**Table 4:** Experiences in living with HIV among Dutch aMASE-study participants (*n* = 247), 2013–2015

	Total		Migrant MSM		Non-migrant MSM		Migrant heterosexual men		Non-migrant heterosexual men		Migrant women	
	(*n* = 247)		(*n* = 77)		(*n* = 115)		(*n* = 21)		(*n* = 14)		(*n* = 20)	
	n	%	n	%	n	%	n	%	n	%	n	%
Disclosure of HIV status to steady partner^a^	118/126	93.7	32/36	88.9	60/62	96.8	9/10	90.0	8/8	100.0	9/10	90.0
Disclosure of HIV status to friends or family	204/246	82.9	63/77	81.8	97/115	84.3	16/21	76.2	12/14	85.7	16/19	84.2
In contact with any NGO’s for HIV support	56/246	22.7	14/77	18.2	34/115	29.6	3/21	14.3	2/14	14.3	3/20	15.0
Ever been discriminated in the Netherlands because of HIV status	40/246	16.3	19/76	25.0	10/115	8.7	3/21	14.3	2/14	14.3	6/20	30.0
Ever been discriminated in the Netherlands because of ethnicity, race or origin	50/116	43.1	30/76	39.5	NA		9/21	42.9	NA		11/19	57.9
Ever been discriminated in the Netherlands because of sexuality	88/191	46.1	31/76	40.8	57/115	49.6	NA		NA		NA	

*MSM* men who have sex with men, *NGO* non-governmental organization, *NA* Not applicable

^a^ Only participants were included who had a steady partner

In univariable analyses (Fig. 2c), migrant MSM (OR:3.50, 95%-CI:1.52–8.03) and migrant women (OR:4.50, 95%-CI:1.42–14.29) were more likely to report ever been discriminated in the Netherlands because of their HIV-status than non-migrant MSM. Other outcomes on experiences in living with HIV did not differ significantly between groups.

Comparing outcomes of experiences in living with HIV by region of birth, MSM born in a region other than sub-Saharan Africa, Latin America/Caribbean or Europe were less likely to be in contact with an NGO for HIV support than non-migrant MSM (OR:0.11, 95%-CI:0.39–3.13). MSM born in sub-Saharan Africa (OR:3.50, 95%-CI:0.33–36.86), Latin-America/Caribbean (OR:1.97,95%-CI:0.49–7.93), another country in Europe (OR:4.50, 95%-CI:1.63–12.42) or another region (OR:3.71, 95%-CI:1.19–11.52) were more likely to have experienced HIV discrimination in the Netherlands than non-migrant MSM, although this effect was not statistically significant for MSM from sub-Saharan Africa and Latin-America/Caribbean. Other outcomes on experiences in living with HIV did not differ significantly between region of birth among MSM.

When adjusting for age, outcomes on access to testing and healthcare pre-diagnosis, access to treatment and ongoing HIV care and experiences in living with HIV yielded comparable results.

## Discussion

This study, focusing on migrant and non-migrant persons recently diagnosed with HIV living in the Netherlands, found disparities in access to HIV prevention, testing and care and experience of HIV-related discrimination by migrant status but also by sexual orientation.

Previous HIV testing and late-stage HIV infection diagnosis did not differ between migrant and non-migrant MSM. However, migrant and non-migrant heterosexual participants were less likely to have had an HIV test before their HIV diagnosis and were more often diagnosed with a late-stage HIV infection than non-migrant MSM, indicating they are facing barriers in accessing HIV testing services. The finding that almost all participants visited a healthcare facility in the 2 years before HIV diagnosis but only in 40% an HIV test was discussed suggests that testing opportunities are being missed, as has been demonstrated at the European level [9]. In line with our findings, another study in the Netherlands found that HIV testing was often not discussed during GP consultations prior to HIV diagnosis [12]. These data suggest that increased provider-initiated testing, especially at the GP but also during hospital admissions, in dental or mental health facilities, is needed to increase earlier HIV diagnosis, especially among heterosexuals. Provider-initiated HIV testing in such settings is particularly important as data from the aMASE community survey showed that low risk perception is one of the main barriers to HIV testing among both heterosexual migrants and migrant MSM [8].

Our data show that the majority of migrant (90%) and non-migrant (84%) MSM had a negative HIV test before their HIV diagnosis. However, previous estimations of the aMASE study data showed that a considerable proportion of HIV-positive migrant MSM in Europe and the Netherlands acquired their HIV infection postmigration [10]. Therefore, improving early access to behavioural and biomedical HIV prevention interventions (i.e., pre- and post exposure prophylaxis [PEP and PrEP]) among HIV-negative migrant MSM is important. As a first step increasing awareness of biomedical interventions among migrant MSM is necessary as we show that PEP awareness was significantly lower among migrant MSM than among non-migrant MSM. The latter might be indicative for lower levels of awareness of PrEP and other HIV prevention strategies, such use of (free) condoms, which we did not measure.

In our study approximately one-fifth of all migrants experienced difficulties accessing healthcare in the Netherlands, which was significantly higher than among non-migrant MSM. Most reported difficulties were uncertainty about entitlement to healthcare and language barriers. These barriers are not specific to HIV-related healthcare services and have been described widely in other studies regarding access to healthcare among migrants [13]. Although almost all participants were registered at a GP and migrants living with HIV in the Netherlands experience less difficulties accessing healthcare than migrants in some other countries in Europe [9, 14], these structural barriers in the access to care for migrants need to be addressed. In the Netherlands, all residents (including asylum seekers and refugees) are entitled to a basic health insurance package which includes the bulk of essential healthcare (including care provided by a GP), medications and medical aids [15]. For undocumented migrants who lack the resources to pay for healthcare, systems are in place to reimburse medical costs [16]. Knowledge about these rights to care should be improved. Also, healthcare systems need to become more migrant-friendly, e.g., overcoming language and cultural barriers in service delivery, improving the culture competencies of health workers and organisations and improving health literacy (i.e., the degree to which an individual has the capacity to obtain, communicate, process, and understand basic health information and services to make appropriate health decisions) [13, 17, 18]. In order to decrease barriers for migrant communities, alternative options for HIV prevention, testing and care besides traditional healthcare settings should also be explored, for example through community outreach, expanding HIV support via NGO’s for groups currently not reached, and the use of HIV self-tests [19–21]. As our data show about 75% of heterosexual migrant visited a religious service, partnerships with and community outreach through religious services could be utilized to increase HIV testing.

In regard to access to HIV treatment and care, overall cART usage was high (86%) and, although 14% reported difficulties taking HIV medication on a regular basis, it was encouraging that self-reported cART adherence was high, with no significant differences between groups. However, we found that migrant heterosexual men and women more often reported missing an appointment at their HIV clinic because of travel expenses than non-migrant MSM. This difference is most likely related to a lower socioeconomic status and these results are consistent with European data [8, 9]. As missing clinical appointments might lead to suboptimal HIV care and treatment, efforts should be made to discuss the costs of travel with HIV-positive patients not showing up for their appointments and opportunities to overcome such barriers should be explored. Furthermore, we found a high proportion of migrant heterosexual men and migrant women were earning less than minimum wage and experienced moderate/severe household hunger. The potential impact of poverty in this group on access to care and quality of life should be further explored.

Furthermore, our study shows that about a quarter of migrant MSM and migrant women had experienced HIV discrimination in the Netherlands. Previous studies have shown that HIV stigma and related HIV discrimination is a major barrier to accessing prevention, care, and treatment services and can negatively impact social relationships, the psychological wellbeing of people living with HIV/AIDS and labour participation [22–28]. As we did not ask participants about the context of HIV discrimination, it is unclear if discrimination took place at the structural, individual or community level [22, 27]. As showed that the psychological impact of stigma varies by social setting [26] and interventions to tackle HIV discrimination differ per setting [27], collecting data regarding the setting in which HIV discrimination took place in future studies is important in order to develop effective interventions. We also observed high levels of ethnic discrimination among migrants (43%) and, among MSM, high levels of discrimination due to sexuality (46%). These proportions are worrisome and interventions are urgently needed to limit the potential impact on quality of life and access to care.

The main strength of our study is the comparison of a rich set of data between HIV-positive migrants and non-migrants living in the same country, data not available in many studies evaluating access to services among migrants [8, 9]. However, some limitations need to be addressed beside the limitations of the aMASE study described elsewere [7, 9]. First, due to low numbers, non-migrant women were not included in our analyses and therefore we were not able to compare outcomes between migrant and non-migrant women. Second, in our additional analyses we only corrected for age and not for socio-economic characteristics such as educational and income level. The small numbers in our study limited us to perform multivariable analyses in which variables other than age were included. Additionally, variables on socio-economic status were highly collinear with migrant status, making it difficult to tease apart the independent association of migrant status on the various outcomes with respect to socio-economic status. Third, although the survey was comprehensive, detailed information to assess determinants of underlying barriers that limit access to HIV related health services was not possible. Fourth, our results might not be generalizable to other countries/settings as they might serve different populations and migrant groups, and have different health systems. Also, the response rate of a study might affect the generalizability. The response rate in our study was 60%, which is relatively high for a study covering sensitive topics such as HIV, sexuality and discrimination and focussing on migrants. However, there might be selection bias as non-respondent analyses showed that migrants from Latin America/Caribbean and women and heterosexual men were less likely to participate.

## Conclusions

In conclusion, we observed disparities in access to and use of HIV-related health services and experiences in living with HIV by migrant status but also by sexual orientation. To make services more accessible and to ensure timely HIV prevention, diagnosis and care, interventions need to be tailored according to the individual. Our data suggests heterosexual men and women may particularly benefit from improved access to HIV testing (e.g. through provider-initiated testing), while migrant MSM may benefit from improved access to HIV prevention interventions (e.g., PrEP).

## Data Availability

The data that support the findings of this study are available from the corresponding author (jbil@ggd.amsterdam.nl) and the aMASE study group but restrictions apply to the availability of these data, which were used under license for the current study, and so are not publicly available. Data are however available from the authors upon reasonable request and with permission of the aMASE study group.

## Abbreviations

AIDS: Acquired immunodeficiency syndrome
aMASE study: Advancing Migrant Access to health Services in Europe Study
ATHENA HIV cohort database: AIDS Therapy Evaluation in the Netherlands HIV cohort database
cART: Combination antiretroviral therapy
HIV: Human immunodeficiency virus
IQR: Interquartile range
MSM: Men who have sex with men
NGO: Non-governmental organization
OR: Odds ratio
PEP: Post-exposure prophylaxis
PrEP: Pre-exposure prophylaxis
